# Identifying and Validating Genes with DNA Methylation Data in the Context of Biological Network for Chinese Patients with Graves' Orbitopathy

**DOI:** 10.1155/2019/6212681

**Published:** 2019-03-14

**Authors:** Ting-Ting Shi, Lin Hua, Zhong Xin, Yu Li, Wei Liu, Yi-Lin Yang

**Affiliations:** ^1^Department of Endocrinology, Beijing Tongren Hospital, Capital Medical University, Beijing, China; ^2^Department of Mathematics, School of Biomedical Engineering, Capital Medical University, Beijing, China; ^3^Physical Examination Department, Beijing Tongren Hospital, Capital Medical University, Beijing, China

## Abstract

**Aim:**

This study investigated the association of DNA methylation with Graves' orbitopathy (GO) incidence through a combined analysis in the context of biological network to identify and validate potential genes for Chinese patients with GO.

**Methods:**

A genome-scale screening of DNA methylation was performed on the peripheral blood sample of six patients with GO and six controls. After extracting differentially methylated regions (DMRs), the study focused on two classes of genes with obviously different methylation levels: low methylated genes (LMGs) and high methylated genes (HMGs). Mutual information was applied to construct LMG- and HMG-regulated networks, and the top 10 LMGs and HMGs were extracted based on the topological properties. Then, 9 candidate genes were extracted to validate their association with GO in an expanded population (48 patients with GO vs. 24 normal controls) using single-cell methylation sequencing.

**Results:**

In the LMG-regulated network, some LMGs displayed a higher degree, such as HIST1H2AL, EFCAB1, and BOLL. Similarly, in the HMG-regulated network, some HMGs, such as MBP, ANGEL1, and LYAR, also showed a higher degree. For validation using an enlarged population, BOLL still displayed the lower methylation level whereas CDK5 and MBP still displayed the higher methylation level in patients with GO in the multivariable logistic regression analysis adjusted by age and gender (*P* < 0.01).

**Conclusions:**

BOLL, CDK5, and MBP are potential genes associated with GO. This study was novel in clinically investigating the relation of these genomic loci with GO. The findings might provide new insights into understanding this disease.

## 1. Introduction

Graves' orbitopathy (GO) or thyroid-associated ophthalmopathy is an inflammatory orbital disease of autoimmune origin with the potential to cause severe functional and psychosocial effects. GO often occurs with an abnormal thyroid function, usually hyperthyroidism. It presents one of the most difficult challenges in the clinical practice of endocrinology and ophthalmology, largely because available treatments do not target pathogenic mechanisms [1].

A previous study found that DNA methylation (DNAm) differences were associated with GO through a genome-wide DNAm analysis in the peripheral blood. Several genomic loci were identified to have significant differences in methylation patterns associated with GO incidence [2]. In fact, some findings indicated that DNAm was primarily a function of promoter CpG content, which results in a constitutive hypo- or hypermethylated state. Recently, a large number of studies are investigating the relationship between DNAm levels and the expression or functions of associated genes [3]. Housekeeping functions are significantly overrepresented in the low methylated genes (LMGs), whereas specific functional characteristics of more differentiated or highly regulated cells are significantly overrepresented in the high methylated genes (HMGs) [4, 5]. In addition, single DNAm data analysis has yielded limited results. Hence, a substitute integrated data analysis should be performed to gain more benefits in biomarker discovery.

This study focused on two types of genes (LMGs and HMGs), constructed LMG- and HMG-regulated networks based on mutual information [6], and calculated their topological properties. These LMGs and HMGs with prominent topological properties were taken as candidate genes that might contribute to the gene network. Then, some candidate genes were extracted to validate their association with GO in an expanded population using single-cell methylation sequencing. The findings might help understand the potential relationships between the DNAm level of some certain genes and their regulation in patients with GO.

## 2. Materials and Methods

### 2.1. Study Participants

In a previous study, DNA was obtained from six Chinese patients with GO and six age-matched controls who had normal thyroid function and no clinical manifestations of eye diseases. Then, a reduced representation bisulfite sequencing (RRBS) assay was performed [2]. Next, an enlarged sample of 48 patients with GO and 24 normal controls was selected to validate the association of extracted candidate genes with GO, and a targeted bisulfite sequencing assay was performed. The validation sample included 34 male and 38 female participants (aged 21 to 71 years, average age 45.1 ± 9.9 years). No significant differences in age (*P* = 0.194), gender (*P* = 0.867), and other clinical parameters were found between cases and controls (Table 1).

The diagnosis of GO was based on the EUGOGO consensus [7]. The clinical activity score (CAS) was recorded using the EUGOGO patient form. TRAb was measured using commercially available electrochemiluminescence assays based on the M22 monoclonal antibody, with a normal range < 1.75 U/L (Roche Diagnostics GmbH). CT or MRI was used to exclude any orbital space-occupying disease such as tumor or extraocular myositis. None of the patients received any immunosuppressive therapy or radiotherapy previously. The study was conducted with the approval from the Ethics Committee of Beijing Tongren Hospital, Capital Medical University. Written informed consent was obtained from each participant.

### 2.2. RRBS

The RRBS assay was performed [8]. 10 G of 2 × 150 bp paired-end raw data was generated from each sample using the Illumina HiSeq 2500 platform. The adapter-trimmed and quality-filtered clean reads were aligned to the bisulfite-converted reference genome hg19, and the methylation levels of cytosine in CpG, CHG, and CHH context were calculated separately. These levels were used to calculate the methylation levels of CPGI, gene, and TSS regions. CpG sites covered with at least five reads were used for subsequent analysis. The detailed description is provided in a previous study [2].

### 2.3. Targeted Bisulfite Sequencing Assay

The DNAm level was analyzed by MethylTarget (Genesky Biotechnologies Inc., Shanghai, China), an NGS-based multiple-target CpG methylation analysis method. Specifically, the genomic regions of interest were analyzed and transformed into bisulfite-converted sequences using geneCpG software. Polymerase chain reaction (PCR) primer sets were designed from bisulfite-converted DNA using the Methylation Primer software [9].

Genomic DNA (400 ng) was subjected to sodium bisulfite treatment using the EZ DNA Methylation-Gold Kit (Zymo Research) according to the manufacturer's protocols. Multiplex PCR was performed with an optimized primer set combination. A 20 *μ*L PCR reaction mixture was prepared for each reaction, including 1× reaction buffer (TaKaRa), 3 mM Mg^2+^, 0.2 mM dNTP, 0.1 *μ*M of each primer, 1 U HotStarTaq polymerase (TaKaRa), and 2 *μ*L of template DNA. The cycling program was 95°C for 2 min, 11 cycles of 94°C for 20 s, 63°C for 40 s with a decreasing temperature step of 0.5°C per cycle, 72°C for 1 min, followed by 24 cycles of 94°C for 20 s, 65°C for 30 s, 72°C for 1 min, and, finally, 72°C for 2 min.

PCR amplicons were diluted and amplified using indexed primers. Specifically, a 20 *μ*L mixture was prepared for each reaction, including 1× reaction buffer (NEB Q5), 0.3 mM dNTP, 0.3 *μ*M of F primer, 0.3 *μ*M of index primer, 1 U Q5 DNA polymerase (NEB), and 1 *μ*L of diluted template. The cycling program was 98°C for 30 s, 11 cycles of 98°C for 10 s, 65°C for 30 s, 72°C for 30 s; and, finally, 72°C for 5 min. PCR amplicons (170–270 bp) were separated by agarose electrophoresis and purified using the QIAquick Gel Extraction Kit (Qiagen).

Libraries from different samples were quantified and pooled together, followed by sequencing on the Illumina MiSeq platform according to the manufacturer's protocols. Sequencing was performed with a 2 × 300 bp paired-end mode. The quality control of sequencing reads was performed using FastQC. Filtered reads were mapped to the genome using BLAST. After recalibrating reads with USEARCH, methylation and haplotype were analyzed using the Perl script.

### 2.4. Statistical Analysis

The methylKit [10] and eDMR [11] software were used to extract differentially methylated regions (DMRs) in genomic regions. A *P* value < 0.05 indicated a statistically significant difference. For these identified DMRs, the focus was primarily on two classes of genes in patients with GO: LMGs and HMGs. In this study, LMGs were defined as DMRs with fold_change < 5th percentile whereas HMGs were defined as DMRs with fold_change > 95th percentile. For LMGs and HMGs, mutual information (MI) was applied to construct LMG- and HMG-regulated networks, respectively. MI has attractive information-theoretic interpretations and can be used to measure nonlinear associations. Therefore, it is often used as a generalized correlation measure. In practice, inferring networks using MI has been shown to be an effective strategy. The minet package of R software (http://www.bioconductor.org) was used to implement this analysis. Then, the corresponding topological properties of LMG- and HMG-regulated networks were calculated using the Cytoscape software (http://www.cytoscape.org/). The network topological properties of each node (LMGs or HMGs) were described simply as follows: (1) degree: the degree of the node in the network is the number of connections it has to other nodes. (2) Betweenness, *B*(*v*): *B*(*v*) can be calculated from the number of shortest paths *σ*_*st*_ from nodes *s* to *t* going through *v* [12]:

(1) $$\begin{array}{c} B\left(v\right)=\underset{s\neq v,s\neq t,v\neq t\in V}{\sum }\sigma_{st}\left(v\right)/\sigma_{st}. \end{array}$$

(3) Closeness: the closeness of a node is a measure of centrality in a network, calculated as the sum of the length of the shortest paths between the node and all other nodes in the graph.

For nodes (LMGs or HMGs) with a higher degree, betweenness and closeness mean that these genes link more genes and may be highly conserved in maintaining the housekeeping biological functions of cells. Thus, they are potential genes associated with the disease. Accordingly, for LMG- and HMG-regulated networks, the top 10 genes with outstanding topological properties were extracted as candidate genes.

For candidate genes obtained from the analysis of topological properties, the Pearson and Spearman correlations were applied to show the association between their methylation levels and clinical phenotype (age, SBP, DBP, BMI, duration, CAS, and TRAb). These significant associations with *P* < 0.05 were shown in the network along with their correlation coefficients.

The independent-sample *t* test, univariate logistic regression, and multivariate logistic regression adjusted by age and gender were performed to validate the DMRs. A *P* value < 0.05 indicated a statistically significant difference.

### 2.5. Validation

Some candidate genes were extracted to validate their association with GO in the expanded population by combining the aforementioned analysis and the previous findings. According to the genome-wide DNAm data of the BOLL gene, the difference in means was 12.86 and the standard deviation of the two groups was 3.30 and 10.76, respectively. Further, 10 case-control sets (1 case to 1 control) with a 90% power and 5% significance were needed. Therefore, in practice, 48 patients with GO and 24 healthy controls were enrolled using single-cell methylation sequencing.

Published studies were manually searched to see the potential association of top 10 LMGs and HMGs, having outstanding topological properties based on the network analysis, with GO. Genes having associations with GO directly or indirectly were selected as the candidate genes. Considering the limitations of data analysis, some significant genes obtained from a previous study were added as supplementary candidate genes; these genes were also proved to have potential associations with GO directly or indirectly. Finally, the candidate genes were used to perform validation analysis in the expanded population.

**Figure 1:**
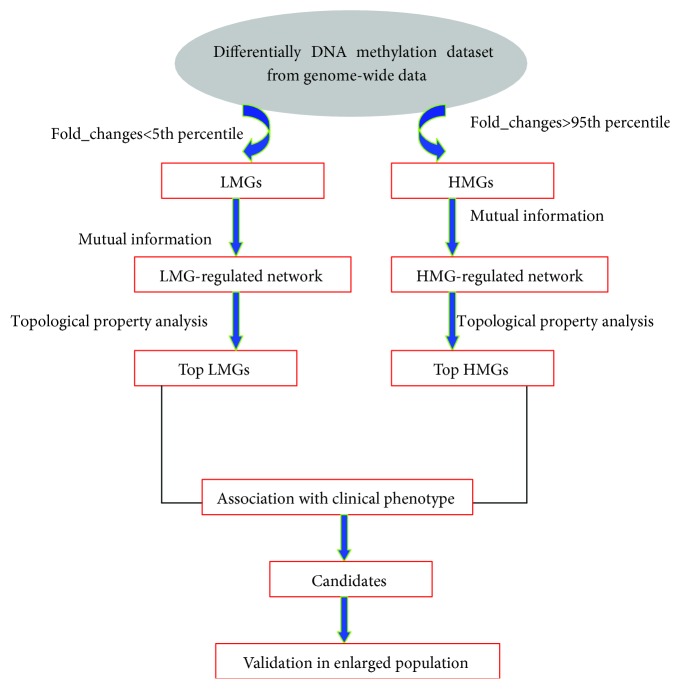
Flowchart of this study. First, LMGs as DMRs with fold_change < 5th percentile and HMGs as DMRs with fold_change > 95th percentile were extracted. Second, LMG- and HMG-regulated networks were constructed based on mutual information. Third, the top 10 LMGs and HMGs were extracted as candidate genes. Fourth, for these candidate genes, the Pearson and Spearman correlations were performed to show the association between their methylation levels and clinical phenotype in the network. Finally, some candidates were selected to validate their association with GO in an enlarged population using single-cell methylation sequencing.

The flowchart of the study is shown in Figure 1.

## 3. Results

### 3.1. Identification of DMRs

According to the criterion of *P* < 0.05, 1583 DMRs were identified. After removing these regions obtained from the missing samples, 841 differentially methylated sites were extracted [2] and used for further analysis. LMGs were defined as those DMRs with fold_change < 5th percentile whereas HMGs were defined as those DMRs with fold_change > 95th percentile. According to this criterion, 42 LMGs (fold_change < 0.393) and 42 HMGs (fold_change > 2.193) were selected for further analysis.

### 3.2. Network Construction and Analysis of Topological Properties

For LMGs and HMGs, MI was applied to construct LMG- and HMG-regulated networks, respectively. Pairs with MI > 0 were taken as interaction pairs and kept for constructing the network. The LMG-regulated network included 316 pairs, and the HMG-regulated network included 322 pairs. The average MI of the HMG-regulated network was 0.032, which was lower than that of the LMG-regulated network (0.378). Thus, LMGs were inclined to interact with other LMGs.

**Figure 2:**
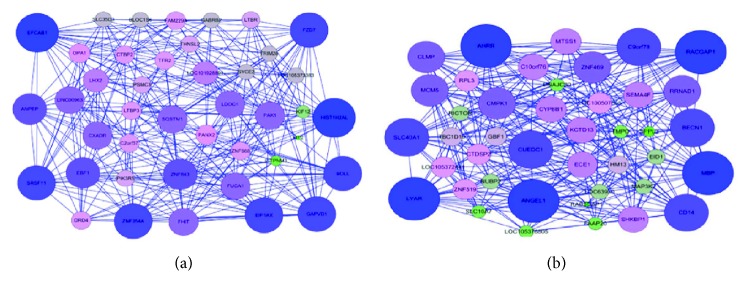
(a) LMG-regulated network. (b) HMG-regulated network. In these two networks, the node with a larger size and deeper color indicates a gene with a higher degree.

Moreover, the topological properties of the two networks were also calculated; the results are shown in Table 2. The LMG-regulated network had a higher network heterogeneity and network centralization compared with the HMG-regulated network, implying that stronger links were inclined to present in LMGs than in HMGs. Then, three topological properties (degree, betweenness, and closeness) of each LMG and HMG involved in the networks were calculated. In the LMG-regulated network, some LMGs displayed a higher degree, such as HIST1H2AL, EFCAB1, and FZD7 (Figure 2(a)). Similarly, in the HMG-regulated network, some HMGs, such as MBP, ANGEL1, and LYAR, also showed a higher degree (Figure 2(b)).

**Figure 3:**
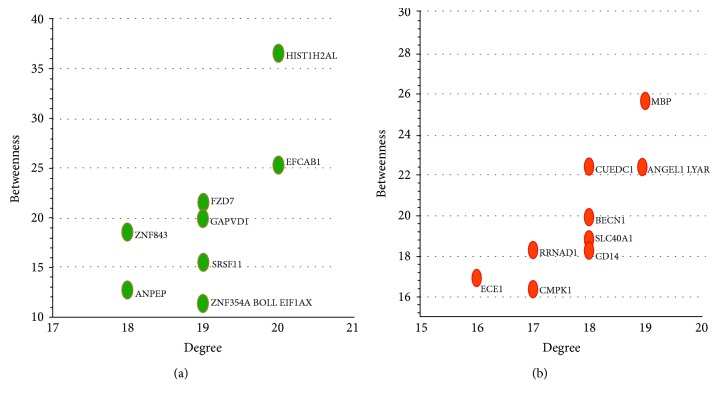
(a) Top 10 LMGs with the highest degree and betweenness. (b) Top 10 HMGs with the highest degree and betweenness.

The top 10 LMGs and HMGs with outstanding topological properties were extracted as candidate genes. Considering the closeness of these candidate genes, only the two-dimensional graph based on two topological properties (degree and betweenness) was displayed (Figure 3). Some HMGs, such as MBP, an immunodominant epitope of myelin basic protein that binds to the major histocompatibility complex haplotype HLA-DR2, are widely implicated in the pathogenesis of autoimmune diseases [13]. As another HMG, the BECN1 complex is proved to be necessary for the fusion of autophagosomes and endosomes with lysosomes [14]. A recent study found that USP19 affected the ubiquitination of BECN1. Therefore, the BECN1-USP19 axis is important in the crosstalk between autophagy and antiviral immune responses [15]. Autophagy is necessary for adipogenesis, and the protective effects against autophagy may help in preventing GO [16].

### 3.3. Correlation between the Clinical Phenotypes and the Methylation Levels at the Identified Candidate Loci

**Figure 4:**
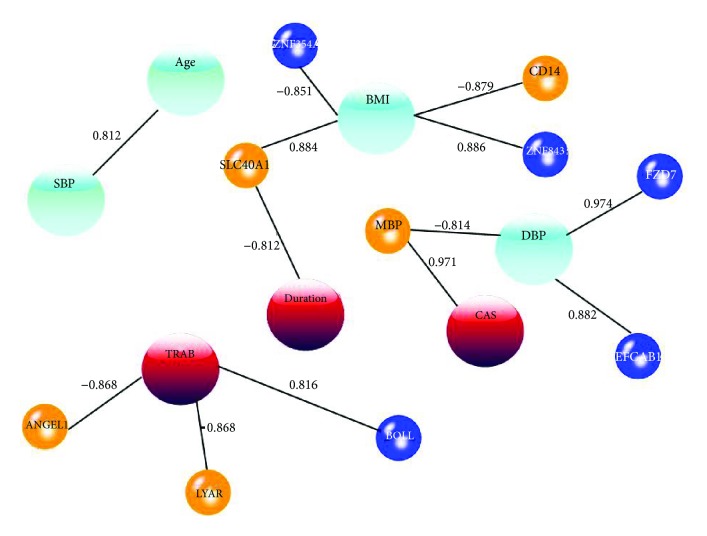
Association between candidate genes and clinical phenotypes in a network. The small orange circles indicate HMGs, whereas the small blue circles indicate LMGs. Age, SBP, BMI, and DBP are general phenotypes shown in large light blue circles. TRAb, duration, and CAS are GO-related clinical phenotypes shown in deep red circles.

For 20 candidate genes obtained from the analysis of topological properties, the Pearson and Spearman correlations were applied to show the association between their methylation levels and clinical phenotype (age, SBP, DBP, BMI, duration, CAS, and TRAb) in the network. These significant associations with *P* < 0.05 were shown in the network along with their correlation coefficients (Figure 4). HMGs at ANGEL1 and LYAR were negatively associated with TRAb, whereas LMGs at BOLL were positively associated with TRAb. In addition, HMGs at MBP were positively associated with CAS.

### 3.4. Validation

**Figure 5:**
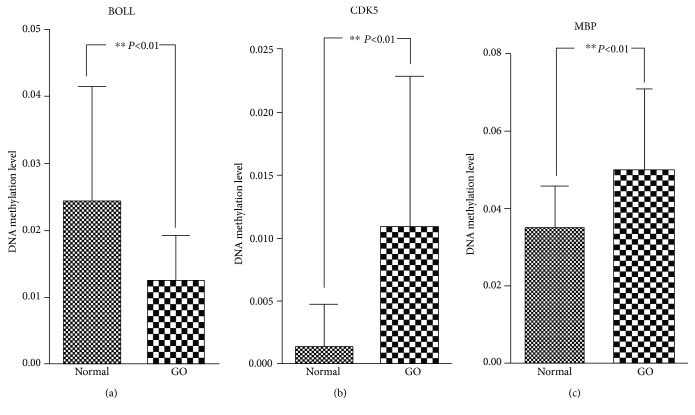
Methylation levels at DMRs between control and patients with GO based on the enlarged population. DMRs: differentially DNA methylated regions.

Some candidate genes were extracted to validate their association with GO in the expanded population by combining the aforementioned analysis and the previous findings. Among the top 10 LMGs and HMGs with outstanding topological properties based on the network analysis, ANGEL1, BECN1, BOLL, CD14, LYAR, and MBP were selected as candidate genes. CDK5, DRD4, and IL17RE, identified as potential genes associated with GO by a previous study, were selected as supplementary candidate genes [2]. The independent-sample *t* test and univariate logistic regression showed that the loci at ANGEL1, BECN1, BOLL, CDK5, IL17RE, LYAR, and MBP were significant DMRs between patients with GO and controls. The multivariable logistic regression adjusted by age and gender revealed that BOLL still displayed the lower methylation level (*P* < 0.01, Figure 5(a)) whereas MBP and CDK5 still displayed a higher methylation level in patients with GO (*P* < 0.01) (Figures 5(b) and 5(c); Table 3).

## 4. Discussion

GO is a condition associated with a wide spectrum of ocular changes, usually in the context of the autoimmune disease, Graves' disease (GD). However, the pathogenesis of GO is not yet fully understood. A genome-wide DNAm analysis revealed the association of DNAm with GO, and several genomic loci were identified [2]. In this study, the potential association between DMRs at some genetic loci, such as BOLL, MBP, and CDK5, was identified and validated in the context of biological network.

Recently, many studies investigated the association between DNAm levels and the expression and functions of associated genes. A study focusing on LMGs and HMGs in the brain tissues found that LMGs were centrally located in the protein-protein interaction network (PPIN) and had higher expression levels compared with the HMGs. In contrast, the HMGs were located in the periphery of the PPIN and formed functional modules of their interacting partners [5]. In this study, as an HMG with prominent topological properties, MBP was validated as the significant DMR between patients with GO and controls using the enlarged samples. It is known that the pathological mechanism for some immune diseases is a breakdown in the peripheral immune tolerance mechanisms. This breakdown allows the activation of MBP-specific CD8 lymphocytes, leading to multiple sclerosis, an inflammatory disease affecting the brain and spinal cord [17]. A study also found that MBP-activated T cells (MBP-T) were important in the damage and repair process of the central nervous system (CNS) [18]. An interactional association between the endocrine and immune systems has been demonstrated under pathophysiological conditions. The binding characteristics of TRa1 to MBP might explain the particular pattern of T3 responsiveness of MBP gene expression during CNS development [19]. A previous finding of a specific hormone-receptor interaction with the MBP promoter region is the direct demonstration of a thyroid hormone-responsive element in a brain-specific gene [20]. CDK5 also displayed higher topological properties in patients with GO. Hence, it can be validated as a DMR in the enlarged population. CDK5 is important in mediating inflammation [21]. A study showed that the deregulated CDK5 promoted oxidative stress by compromising the cellular antioxidant defense system [22].

Most of the LMGs are highly expressed genes and tend to be functionally important genes, such as cancer genes and aging genes. Furthermore, the LMGs tend to be further regulated by microRNAs (miRNAs), implying functional complementation between transcriptional methylation regulation and posttranscriptional miRNA regulation in the human genome [5]. As one of the top 10 LMGs with outstanding topological properties, BOLL is also validated as a significant DMR between patients with GO and controls using the enlarged samples. The function of BOLL protein has not been fully evaluated. However, previous studies have demonstrated its role in spermatogenesis and infertility [23]. Increased cell proliferation and migration were observed in BOLL-transfected cells, suggesting that BOLL functioned as an oncogene in colorectal cancer [23, 24]. Despite no direct evidence indicating the association between BOLL and GO, it has been suggested that BOLL may serve as a novel biomarker for subsequent validation based on molecular biological experiments.

This study had several limitations. First, the smaller sample size might have been insufficient to make conclusive statements regarding the methylation status in patients with GO. Second, the findings might be related to GD itself rather than to GO. Third, the study could not validate the association between methylation levels of candidate genes and clinical phenotype in the expanded population (Supplementary Table 1). This might be due to the individuation of patients with GO and the discretization of their important clinical index such as TRAb. In addition, CAS might not reflect the inflammatory activity of GO, although it is the most widely used indicator [25]. Therefore, the association between genetics and the orbital imaging data should be the focus of future studies to explore the pathogenesis of GO using more samples. Finally, the study only tested the DNAm levels of genes in patients with GO. Increased methylation is known to result in decreased gene expression levels in some diseases [26]. Some recent studies observed that gene expression was regulated by genetic variation via DNAm instead of gene expression affecting DNAm levels [27]. Therefore, the association between the expression level of candidate genes and their methylation levels is important. Future studies should integrate different molecular biology data, including gene expression level, to explore the complex regulation of biomarkers contributing to GO.

In summary, this study identified and validated BOLL, MBP, and CDK5 as potential genes associated with GO by combining the present and previous results. The findings based on the genome-scale screening of DNAm and the single methylation sequencing validation using enlarged samples might provide new insights into understanding this disease and provide new treatment and prevention strategies for Chinese patients with GO. This study was the first clinical investigation of the association of BOLL, MBP, and CDK5 with GO. Further studies are needed to elucidate their precise roles in this disease.

## Figures and Tables

**Table 1:** Characteristics of cases and controls for validation.

	Age (year)	Sex (M/F)	Height (cm)	Weight (cm)	SBP (mmHg)	DBP (mmHg)	GO duration (months)	CAS	TRAb (U/L)
Case	46.0 ± 11.4	23/25	165.9 ± 8.1	68.5 ± 14.4	121.9 ± 9.6	74.0 ± 6.1	9.7	3.0 ± 0.8	4.50 (1.69–9.64)
Control	43.4 ± 5.5	11/13	166.9 ± 6.5	66.0 ± 12.9	121.0 ± 18.1	73.0 ± 18.5			<1.75

CAS: clinical activity score; DBP: diastolic blood pressure; SBP: systolic blood pressure; TRAb: thyrotropin receptor antibody.

**Table 2:** Topological properties of LMG- and HMG-regulated networks.

Topological properties	LMG-regulated network	HMG-regulated network
Number of nodes	42	42
Network density	0.367	0.374
Network heterogeneity	0.229	0.165
Clustering coefficient	0.463	0.458
Network diameter	3	3
Network centralization	0.127	0.094
Shortest paths	1722	1722
Characteristic path length	1.646	1.627

HMGs: high methylated genes; LMGs: low methylated genes; number of nodes: the number of nodes in the network; network density: the portion of the potential connections in a network that are actual connections; network heterogeneity: the tendency of a network to contain hub nodes; clustering coefficient: the probability that two vertices connected to the same node are also connected; network diameter: the largest distance between two nodes; network centralization: networks whose topologies resemble a star have centralization close to 1, whereas decentralized networks are characterized by having centralization close to 0; shortest paths: the length of the shortest path between two nodes; characteristic path length: the average shortest path length, which is the expected distance between two connected nodes.

**Table 3:** Significant DMRs at candidate genes between patients with GO and controls based on the enlarged population (48 cases vs. 24 controls).

Target	Chr	Position	Genome position	Type	*P* value (*t*-test)	*P* value (logistic regression)	*P* value (logistic regression adjusted by age and gender)
BOLL	2	200	198651436	CG	0.0042	0.0027	0.011
CDK5	7	93	150754945	CG	2.0852*E*-06	0.0039	0.029
MBP	18	114	74844255	CG	0.0078	0.0115	0.026

DMRs: differentially DNA methylated regions.

## Data Availability

The data used to support the findings of this study are available from the corresponding author upon request.

## Supplementary Materials

Supplementary Materials Supplementary Table 1: associations between clinical parameters and DNAm levels of candidate genes in an expanded population (*P* values).
