# Supplementary material for: Identifying and Validating Genes with DNA Methylation Data in the Context of Biological Network for Chinese Patients with Graves' Orbitopathy
Source: Int J Endocrinol. 2019 Mar 14;2019:6212681. doi: 10.1155/2019/6212681 (PMC6437746; doi:10.1155/2019/6212681)
Supplement: Supplementary Materials — Supplementary Table 1: associations between clinical parameters and DNAm levels of candidate genes in an expanded population (P values). [file 6212681.f1.docx]

Supplementary Table 1. Associations between clinical parameters and DNAm levels of candidate genes in an expanded population (*P* values)

| Clinical parameters | Candidate genes^a^ | | | | | | |
| --- | --- | --- | --- | --- | --- | --- | --- |
|  | ANGEL1 | BECN1 | BOLL | CDK5 | IL17RE | LYAR | MBP |
| Age | 0.824 | 0.995 | 0.943 | 0.330 | 0.268 | 0.723 | 0.996 |
| SBP | 0.641 | 0.361 | 0.084 | 0.413 | 0.577 | 0.210 | 0.738 |
| DBP | 0.050 | 0.413 | 0.481 | 0.732 | 0.309 | 0.353 | 0.663 |
| BMI | 0.526 | 0.238 | 0.795 | 0.115 | 0.299 | 0.500 | 0.807 |
| Duration | 0.387 | 0.588 | 0.400 | 0.926 | 0.841 | 0.460 | 0.715 |
| CAS | 0.479 | 0.888 | 0.977 | 0.652 | 0.738 | 0.919 | 0.518 |
| TRAb | 0.391 | 0.963 | 0.415 | 0.357 | 0.306 | 0.312 | 0.082 |

^a^Candidate genes: Differential methylation candidate genes based on the data analysis of expanded population.

CAS, Clinical activity score; DBP, diastolic blood pressure; SBP, systolic blood pressure; TRAb, thyrotropin receptor antibody.
